# Resource-Efficient Parallelized Random Access for Reliable Connection Establishment in Cellular IoT Networks

**DOI:** 10.3390/s23083819

**Published:** 2023-04-08

**Authors:** Taehoon Kim, Seongho Chae, Jin-Taek Lim, Inkyu Bang

**Affiliations:** 1 Department of Computer Engineering, Hanbat National University, Daejeon 34158, Republic of Korea; 2 Department of Electronics Engineering, Tech University of Korea, Siheung 15073, Republic of Korea; 3 Agency for Defense Development, Daejeon 34186, Republic of Korea; 4 Department of Intelligence Media Engineering, Hanbat National University, Daejeon 34158, Republic of Korea

**Keywords:** internet-of-things, random access, connection establishment, redundancy elimination, resource efficiency

## Abstract

The role of various internet-of-things (IoT) devices responsible for data collection and reporting becomes more important in the era of Industry 4.0. Due to the various advantages (e.g., wide coverage, robust security, etc.), the cellular networks have been continuously evolved to accommodate IoT scenario. In IoT scenario, connection establishment is essential and primary for enabling IoT devices to communicate with centralized unit (e.g., base station (BS)). This connection establishment procedure in cellular networks, random access procedure, is generally operated in a contention-based manner. So, it is vulnerable to simultaneous connection requests from multiple IoT devices to the BS, which becomes worse as the contention participants increase. In this article, we newly propose a resource-efficient parallelized random access (RePRA) procedure for resource-efficiently ensuring reliable connection establishment in cellular-based massive IoT networks. Key features of our proposed technique are twofold: (1) Each IoT device simultaneously performs multiple RA procedures in parallel to improve connection establishment success probability, and (2) the BS handles excessive use of radio resources based on newly proposed two types of redundancy elimination mechanisms. Through extensive simulations, we evaluate the performance of our proposed technique in terms of connection establishment success probability and resource efficiency under various combinations of control parameters. Consequently, we verify the feasibility of our proposed technique for reliably and radio-efficiently supporting a large number of IoT devices.

## 1. Introduction

The next-generation communication system, i.e., 6G, is expected to be emerged around 2030, and it is obvious that further enhanced system performances (e.g., data rate, connectivity density, and latency) compared to 5G will be required to support innovative applications such as autonomous driving car, extended reality (XR), and so on [1,2]. Furthermore, due to the wave of the 4th industrial revolution, the techniques aiming for artificial intelligence (AI) have come to every industry field including communication & networks [3]. Accordingly, the importance of data is further emphasized, and the role of various internet-of-things (IoT) devices responsible for data collection and reporting also becomes more important [4]. Deployment density of IoT devices will unprecedentedly increasing, where the number of connected IoT devices is expected to reach 500 billion by 2030 [5,6].

There are various communication protocols suitable for supporting IoT services such as Advanced Message Queuing Protocol (AMQP) [7], Message Queuing Telemetry Transport (MQTT) [8], LoRa [9], SigFox [10], Narrow Band IoT (NB-IoT) [11], Long Term Evolution (LTE) [12], and 5G New Radio (5G NR) [13]. Among them, the cellular systems (e.g., LTE and 5G NR) have continuously attracted great attention as one of the promising candidates suitable for IoT services due to its tremendous advantages such as wide coverage, robust security, and infrastructure in place [12,13]. However, to exploit cellular services, every mobile terminals including IoT devices should make a radio connection with the centralized unit, i.e., base station (BS) (In LTE and 5G/5G+, the BS is called eNB and gNB, respectively), before the data communications are performed. This connection establishing procedure is called random access procedure in cellular systems [14,15], which is a contention-based protocol, i.e., a variation of multi-channel slotted ALOHA protocol [16]. This implies that the collision problem may arise during the RA procedure which becomes worse as the number of contending participants increases [17]. Since the connection establishment is essential and primary for exploiting cellular services, mitigating the occurrence of collisions during the RA procedure is still of the most importance problem to be solved.

Due to the aforementioned consensus, a number of research groups have made enormous efforts to resolve the collision problem. Resource optimized recursive access class barring (ACB) based mechanism was proposed to regulate surge of simultaneous RA attempts [18], and deep reinforcement learning (DRL) based ACB mechanism was also proposed [19]. To mitigate the occurrence of collisions, spatial group based mechanisms to logically increase the amount of contending resources were proposed [20,21]. Zhen et al. [22] proposed an early collision detection mechanism which enables rapid collision detection and load estimation with low computational complexity, and deep learning based double-contention RA was proposed [23]. In order to resolve collisions, the collision resolution scheme using the backoff procedure which dynamically adjusts the backoff indicator based on the number of backlogged devices was proposed [24], and online control algorithm for backoff interval was proposed [25].

It is worth to note that the successful completion of RA procedure is necessary for connection establishment. In order to improve success probability, the RA parallelization (RAP) scheme was proposed [26], where each IoT device is allowed to simultaneously perform multiple RA procedures in parallel. It was proven that this approach definitely improves the performance (i.e., RA success probability) based on the transmission of multiple redundant signals. This may be effective to achieve such performance improvement since the RAP scheme can provide diversity effect during the RA procedure. However, it is obvious that the performance gain comes from the excessive use of radio resources, which still remains as an open issue to be resolved for practical use. In [27], the RAP scheme was partially extended to provide an access priority functionality during the RA procedure, but the problem from the perspective of radio resource efficiency was not also handled.

In this article, we newly propose a resource-efficient parallelized random access (RePRA) technique for resource-efficiently ensuring reliable connection establishment in cellular-based massive IoT networks. We perform simulations to evaluate the performance of our proposed technique from the viewpoint of connection establishment success probability and resource efficiency.

The main contributions of our paper can be summarized as follows:

- We provided meaningful insights regarding how the RA procedure should be evolved considering a scenario where multiple RAs in parallel are available.
- Especially, we newly proposed two redundancy elimination mechanisms to overcome shortcoming (i.e., waste of radio resources) in such a scenario, and, thus, we can achieve the improved radio resource efficiency as well as the improved connection establishment success probability.
- Through extensive simulations, we verified the validity of our proposed technique with various numerical results, and also verified the feasibility of our proposed technique for practical use with the advanced physical-layer techniques not that complicate to be implemented.

The rest of this paper is organized as follows. In Section 2, we provide a brief overview on the random access parallelization (RAP) technique. In Section 3, we describe our system model. In Section 4, we propose a resource-efficient parallelized random access (RePRA) technique and describe it in detail. In Section 5, we evaluate the performance of the proposed technique and provide numerical results. Finally, we draw conclusions in Section 6.

## 2. Brief Review on Random Access Parallelization Technique

In this section, we briefly introduce the RA parallelization (RAP) technique proposed in [26]. The RAP technique can be interpreted as a generalized version of the RA procedure adopted in commercial cellular systems such as LTE/5G [14,15]. The primary difference of the RAP technique from the conventional RA procedure is a relaxation on the number of preambles transmitted in the first step of the RA procedure. To be specific, the RAP technique allows each device to simultaneously transmit multiple RA preambles in the first step of the RA procedure, instead of transmitting a single preamble. Accordingly, each device can significantly mitigate the occurrence of RA failures. The RAP technique also consists of 4-steps of handshaking, and the summarized descriptions on each of steps are as follows:

- (Step 1) Multi-preamble transmissions
  Each IoT device randomly selects multiple *k* different preambles (i.e., k≥1) among a set of available RA preambles, and transmits them simultaneously on the physical RA channel (PRACH) to the BS. (Note that the preambles are a set of commonly shared signals, which are used for contending purpose in this step [13]. Each BS generally configures 64 preamble signatures (i.e., signals) using Zadoff-Chu sequence representing excellent auto/cross-correlation properties [28]).
- (Step 2) Random access responses
  The BS detects which preambles are active. Note that the BS can only detect the existence of signals during the detection phase (e.g., energy detection), and does not know which IoT devices sent those of signals. In response to the detected preambles, the BS broadcasts random access response (RAR) messages, each of which consists of the detected preamble index and an uplink grant (UG), where the actual message transmission occurs in the subsequent step. Each IoT device attempts to identify *k* RAR messages since it triggers the RA procedure with *k* different preambles in the previous step. Accordingly, each IoT device compares the preamble index contained each of RAR messages with *k* preamble indices used in Step 1 to identify the destination of each of RAR messages.
- (Step 3) Multiple scheduled message transmissions
  In this step, each IoT device generates multiple *k* copies of the scheduled message (i.e., *k* connection request messages), and transmits each message on each of the assigned uplink resources. Since the messages containing the identical information are sent *k* times redundantly, this can significantly mitigate the occurrence of the event that the entire messages experience the unexpected resource collisions at the same time.
- (Step 4) Contention resolution
  The BS echoes the identifiers of IoT devices, whose transmitted scheduled messages are successfully decoded without any collisions. If each IoT device receives the correct acknowledgement (ACK) message, then it regards the RA attempt as a success. This implies that the connection with the BS is successfully established. Otherwise, the device considers that the RA attempt fails and connection is not established yet. In this case, each IoT device reattempts the RA procedure at the next-available RA opportunity (i.e., PRACH) after performing a back-off.

## 3. System Model

**Figure 1:**
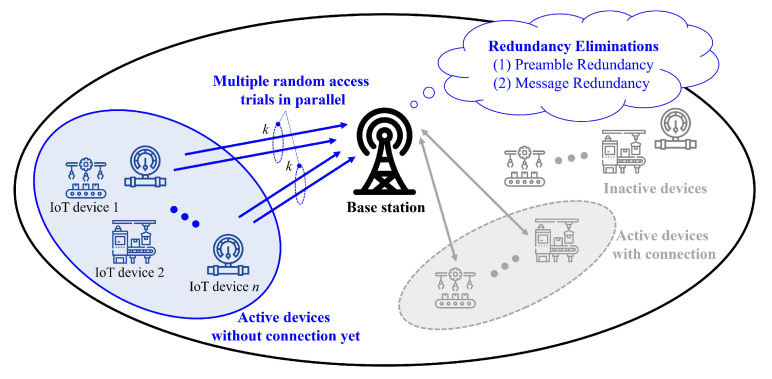
System model: A number of IoT devices are attempting connection establishment with the BS. Note that inactive IoT devices and active IoT devices already have connection with the BS are out of interest in our system model.

We consider a single cell scenario, where a number of IoT devices are deployed within a cell coverage as shown in Figure 1. Generally, each IoT device may be in inactive status, but it transits to active status when it has packets to transmit to the base station (BS) [21]. To communicate with the BS, each active IoT device should establish a connection with the BS by performing random access (RA) procedure. It attempts its RA at the next-available RA occasion (or, equivalently, PRACH), which is periodically configured. In this article, we focus on a specific single RA occasion to take an in-depth look for the effect of our proposed technique on the overall RA performance.

Let *n* denote the number of active devices which are currently participating in the RA procedure at the RA occasion of interest. ( Considering sparse activity of IoT devices, the period of PRACH (e.g., 10 ms), the order of *n* becomes at most tens [26]). Note that *n* does not refer to the total number of IoT devices deployed in a cell coverage. Note that some devices among *n* devices may be in reattempting their RAs due to the previous RA failure.

Suppose that each IoT device can simultaneously transmit multiple RA preambles in Step 1 after randomly selecting multiple different preambles. Unlike the conventional RA scheme [15,29], the number of preambles transmitted at the same time is now relaxed, and this implies each IoT device can proceed multiple RA procedures in parallel. To be specific, *k* represents the number of preambles that can be sent by each of devices at the same time, where k≤M. Here, *M* represents the total number of available RA preambles configured by the central unit, i.e., BS.

## 4. Resource-Efficient Parallelized Random Access for Reliable Connection Establishment

In this section, we propose a resource-efficient parallelized random access (RePRA) technique for resource-efficiently ensuring reliable connection establishment. First of all, we present two redundancy elimination mechanisms which are the core technologies of our proposed RA technique to improve the success probability as well as resource efficiency. Thereafter, we describe our proposed technique with detailed explanations on the overall procedure.

### 4.1. Observations & New Opportunities

Our proposed technique is motivated from the RA parallelization (RAP) scheme proposed in [26]. It deliberately introduces redundancy to the RA procedure, and, thus, it can significantly improve the RA success probability based on the diversity effect achieved during the RA procedure. However, this brings about a side effect that the radio resource may be inefficiently utilized. In summary, even though the RAP scheme in [26] may be effective to provide reliable connection establishment functionality, the efficient management of radio resource should be further elaborated for practical use. If we take a closer look at the potential situation when multiple transmission of redundant signals during the RA procedure is allowed, we can find the following two important observations:

- All the signals (e.g., preambles in Step 1) sent by a certain IoT device experience the same wireless channel, and thus, the power delay profiles (PDPs) of those signals captured at the BS will show almost the same characteristic if they all are exclusively used by the corresponding device.
- When each IoT device redundantly transmits the identical messages (i.e., connection request messages) in parallel, the BS can cancel out redundant messages if there exists at least one successfully decoded message (The fundamental principle may be the same with the successive interference cancellation, but the duplicated message should be found somewhere in other radio resources [30]).

From the observations, we can now newly introduce two redundancy elimination mechanisms which can be incorporated with the RA procedure without significant modifications: preamble redundancy elimination (PRE) and message redundancy elimination (MRE) mechanisms. Note that those mechanisms can be applied to any RA procedure which considers a scenario that multiple RA attempts in parallel are available, where redundant messages are necessarily transmitted.

### 4.2. Preamble Redundancy Elimination

Preamble redundancy elimination (PRE) is a mechanism that the BS avoids responding to the entire preambles that are determined to be sent from the same IoT device. To be specific, if there exist multiple detected preambles showing similar PDP, then the BS transmits only a single representative RA response (RAR) message rather than sending the entire multiple responses. Consequently, redundant subsequent steps (i.e., Step 2 ∼ Step 4) can be efficiently avoided while guaranteeing the same RA success probability. Note that this mechanism is effective to improve the resource efficiency only regardless of the improvement of success probability.

**Figure 2:**
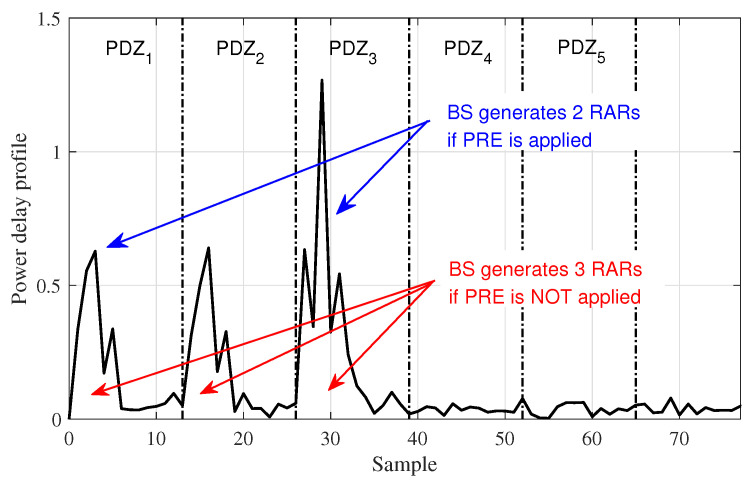
An example of preamble detection result and expected responses according to whether the transmission of multiple preambles is allowed or not.

Figure 2 shows an example of preamble detection result and expected responses according to whether the transmission of multiple preambles in Step 1 is allowed or not. To be specific, since the signals captured in preamble detection zone (PDZ) 1 and PDZ 2 have show similar PDP, the BS can regard that a certain device sent at least two signals at the same time.

### 4.3. Message Redundancy Elimination

Message redundancy elimination (MRE) is a mechanism that the BS cancels out the successfully decoded message (i.e., connection request message) from other radio resources where any messages cannot be decoded due to collision. In other words, there may be originally undecodable messages due to collision, but this mechanism can give another opportunity to the collided messages to be recovered from the residual signal after removing the known (i.e., decoded) signals. This is mainly effective to directly increase the number of successfully decoded messages, and accordingly the radio resource efficiency can be also slightly improved at the same time. ( It is worth to note that there is the case that redundant messages (i.e., connection request message) at Step 3 do not exist in our proposed technique when preamble redundancy is perfectly removed via the PRE mechanism).

**Figure 3:**
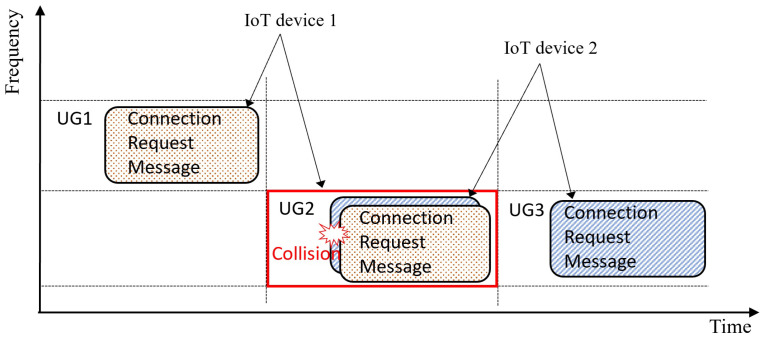
An example of message redundancy elimination when each IoT devices transmit dual connection request messages.

Figure 3 describes a notion of message redundancy elimination with a scenario when both IoT devices transmit dual connection request messages via different radio resources. In this example, the resource collision occurs at the location of UG2. However, since the same message from IoT device 1 can be found at UG1, the message sent by IoT device 2 via UG2 can be recovered after canceling out the message of IoT device 1 from the received signal in UG2.

### 4.4. Overall Procedure

Our proposed technique follows 4-steps of handshaking procedure, and the detailed explanations on each of steps are as follows:

- (Step 1) Multi-preamble transmissions
  Each IoT device triggers its RA procedure by simultaneously transmitting randomly selected multi-preambles to the BS via the PRACH. To be specific, each IoT device randomly selects *k* different preambles from the available preamble set, i.e., $M\in \left\{1,\cdots ,M\right\}$, and transmits them at the same time to the BS. Let $I_{n}$ denote a set of randomly selected preambles of the IoT device *n*, where $I_{n}\subset M$ and $|I_{n}|=k$.
- (Step 2) Preamble redundancy elimination & random access responses
  Note that the role of this step is to generate and send response messages in response to the preamble detection results, but our proposed technique further equips with the redundancy elimination functionality during the preamble detection phase. The BS determines which preambles are active during the preamble detection phase and performs the post-processing procedure, i.e., preamble redundancy elimination. Thereafter, the BS responses to the some of detected preambles by sending RA responses (RARs), each of which includes a detected preamble index and an uplink grant.
  First of all, the BS should obtain power delay profile (PDP) from the received signal via PRACH to detect preambles. To be specific, the PDP can be calculated as $\mathrm{PDP}=|c_{y,r_{zc}}{|}^{2}$, where $c_{y,r_{zc}}$, *y*, and $r_{zc}$ represent the cross-correlation between *y* and $r_{zc}$, the received signal via PRACH, and reference Zadoff-Chu sequence, respectively. The BS separates the PDP into *M* distinct preamble detection zones (PDZs) as $\mathrm{PDP}={⋃}_{m\in M}{\mathrm{PDZ}}_{m}$, where ${\mathrm{PDZ}}_{m}\cap {\mathrm{PDZ}}_{m^{\prime }}=\phi \;\mathrm{for}\;m\neq m^{\prime }$. When a certain level of signal strength is observed in the ${\mathrm{PDZ}}_{m}$, the *m*-th preamble (or, equivalently, preamble *m*) is regarded as active.
  Thereafter, the BS performs PRE mechanism. In detail, the BS compares the similarity between the preamble signals detected in each PDZ. If there exist multiple preambles showing similar PDP, the BS regards them as the preambles sent by an identical device, and avoids generating multiple RARs for them. In this case, instead, the BS generates a single representative RAR rather than generating multiple responses. This procedure enables to avoid redundant steps, and, thus the additional resource consumption in the subsequent steps can be efficiently avoided.
- (Step 3) Multiple scheduled message transmissions
  Each IoT device makes $k^{\prime }$ replicas of its scheduled message (e.g., connection request message) where $k^{\prime }\leq k$ due to the PRE mechanism. Thereafter, it transmits each message on each of the assigned uplink resources, indicated by the UG value contained in each of RARs received in Step 2. This step enables the BS to achieve receive diversity by sending multiple replicas of the original scheduled message. Note that this degrades the resource efficiency in proportion to the level of duplication.
- (Step 4) Message redundancy elimination & contention resolution
  The BS attempts to decode messages received in Step 3 based on Algorithm 1. Particularly, the BS iteratively attempts the message redundancy elimination (MRE) with the successfully decoded messages until no messages are recovered or iteration round reaches *R*, where *R* represents the maximum number of iterations that the elimination procedure is performed. Thereafter, the BS transmits the ACK messages to the IoT devices whose packet is successfully decoded. If each IoT device receives one or more ACK messages, then its RA attempt is regarded as a success.

**Algorithm 1**: Iterative message decoding algorithm**Notations**   $\cdot \;S({\mathrm{UG}}_{i})$: Received signal via UG_*i*_   $\cdot \;\mathrm{DEC}(S\left({\mathrm{UG}}_{i}\right))$: Message decoding attempt with $S({\mathrm{UG}}_{i})$   $\cdot \;R_{u}=\left\{{\mathrm{UG}}_{1},\cdots ,{\mathrm{UG}}_{|R_{u}|}\right\}$: Set of the entire allocated UGs   $\cdot \;R_{s}$: Set of UGs where the messages are successfully decoded   $\cdot \;F({\mathrm{UG}}_{i})$: Set of UGs where the replicas of $S({\mathrm{UG}}_{i})$ are sent**Output**: $R_{s}$    1:  **for** r=1toR **do**2:     $O\leftarrow R_{s}$3:     **for** $i=1\;\mathrm{to}\;|R_{u}-R_{s}|$ **do**4:        **for** $j=1\;\mathrm{to}\;|R_{s}|$ **do**5:           **if** ${\mathrm{UG}}_{i}\in F\left({\mathrm{UG}}_{j}\right)$ **then**6:              **Try** $\mathrm{DEC}\left(S\left({\mathrm{UG}}_{i}\right)-S\left({\mathrm{UG}}_{j}\right)\right)\to \mathrm{Msg}3_{i}$ // MRE7:              **if** success **then** $O\leftarrow O\cup \left\{{\mathrm{UG}}_{i}\right\}$8 :          **end if**9 :       **end for**10:    **end for**11:    $R_{s}\leftarrow O$12: **end for**13: Broadcast the decoding results, $R_{s}$

**Figure 4:**
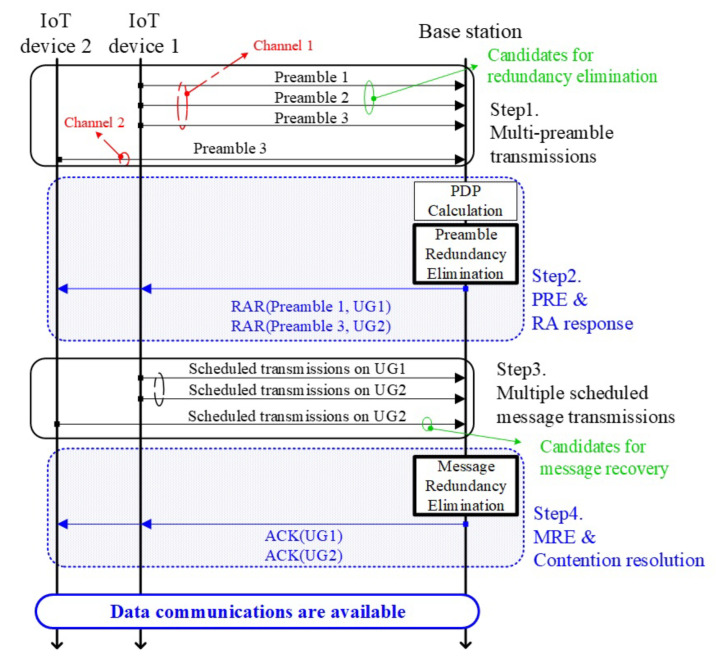
Overall procedure of our proposed technique: Two IoT devices are attempting their RAs with different *k* value (i.e., k=3 and k=1 for IoT device 1 and 2, respectively), and two redundancy elimination mechanisms are applied at the BS.

Figure 4 shows an example that two IoT devices attempt RAs with our proposed technique, and note that the PRE and the MRE mechanisms are newly introduced in Step 2 and Step 4, respectively. For helping clear understanding of the role of each additional mechanism, we assume that two IoT devices use different *k* values, i.e., k=3 and k=1 for IoT device 1 and 2, respectively.

Note that preamble 1, 2, and 3 from IoT device 1 experience the same wireless channel, and preamble 3 is also selected by the IoT device 2. When the BS detects preambles, preambles 1, 2, and 3 will be determined as active and the preambles captured in ${\mathrm{PDZ}}_{1}$ and ${\mathrm{PDZ}}_{2}$ may show almost the same PDP since they experience the same channel. Thanks to the PRE mechanism, the BS generates a single representative RAR instead of generating two RARs from each of them.

Furthermore, preamble 3 is used by two IoT devices at the same time and thus they both transmit their Step 3 messages via UG 2. But, note that the identical message of the IoT device 1 is also transmitted through different radio resource, i.e., UG 1. Since the message received via UG 1 can be decoded successfully, the BS now can remove its effect from UG 2, i.e., S(UG2)−S(UG1), where S(·) represents the signal received via the designated resource, and reattempt to decode message with the residual signal. This can be an obviously new opportunity to recover the device 2’s message from the collision.

Consequently, two IoT devices are able to successfully complete their connection establishments using two uplink resources with our proposed technique. Without considering redundancy elimination mechanisms, only one IoT device can succeed in its RA attempt even the network allocates 3 uplink resources via 3 RARs. From this simple example, we can expect the performance gain from the viewpoint of the resource efficiency as well as the RA success probability.

## 5. Performance Evaluation

In this section, we perform simulations to demonstrate the performance of our proposed technique with a Matlab [31]. Specific simulation parameters are listed in Table 1. Since the amount of radio resources (e.g., preamble) is generally set as a given value, we investigate the effect of other controllable parameters such as the number of connection-requesting active IoT devices per PRACH (i.e., the number of contending participants at a certain time), the number of preambles simultaneously transmitted at Step 1, and the number of iteration rounds during the MRE process, on the performance metrics of interest.

We consider the connection establishment success probability and the resource efficiency as performance metrics. Connection establishment success probability is the probability that an IoT device succeeds in its connection request via RA attempt. Resource efficiency is defined as a ratio of the number of IoT devices which successfully complete their connection request via RA attempt to the amount of assigned uplink resources [17]. This measures how many IoT devices can be radio-efficiently supported using allocated resources. For fair comparison, we consider the conventional RA scheme [14,15], and the RAP scheme [26] as baseline schemes. For all figures, the performance related to the baseline schemes are plotted via both simulation and analysis, and the performance of our proposed technique is plotted via simulation only. In case of our proposed technique, several operating options exist according to the use of each elimination mechanism, which are denoted in the legend of each of figures if needed. Note that we perform 1,000,000 simulations per each numerical setting to reliably measure the performance on average sense.

**Figure 5:**
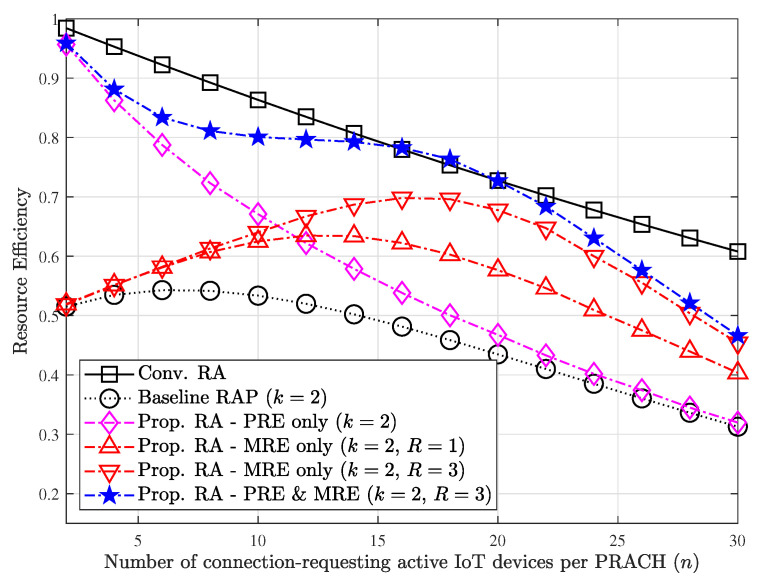
Effect of each of mechanisms on the resource efficiency.

Figure 5 shows the effect of each mechanism incorporated with our proposed technique on the resource efficiency for varying *n* when M=32. Note that the RAP scheme [26] with k=1 operates in the same manner with the conventional RA scheme [14,15]. The purpose of the PRE mechanism is to reduce redundant responses from *k* simultaneous RA attempts, and thus the high performance gain can be found when the load is not severe (i.e., small values of *n*). However, the amount of improvement in resource efficiency decreases as *n* increases since the number of exclusively used preambles may accordingly decrease as *n* increases. On the contrary, the MRE mechanism prefers slightly congested situation, where several devices unintentionally share radio resources due to collisions during Step 3 message transmissions. Thus, the performance gain increases as *n* increases since the occurrence of message collision increases. (The performance gain decreases beyond a certain point as *n* increases due to severe collisions which is hard to be resolved). The performance gain from the MRE mechanism when the value of *n* is small is naturally negligible since there may be few message collisions to be resolved. It is worth to note that the resource efficiency can be improved as the maximum iteration rounds of the MRE process (i.e., *R*) increases since any of recovered messages in each round can be new seed for additional message recovery. Furthermore, the small value of *R* (e.g., R=3) is sufficient enough to exploit the benefit of the MRE mechanism.

Our proposed technique exploiting both redundancy elimination mechanisms exhibits interesting result, where the performance gains from PRE and MRE mechanisms are independently merged. Accordingly, the poor resource efficiency of the RAP scheme can be significantly compensated by our redundancy elimination mechanisms. If the number of contending participants is well controlled, then the resource efficiency can even exceed that of the conventional RA scheme at a certain point.

**Figure 6:**
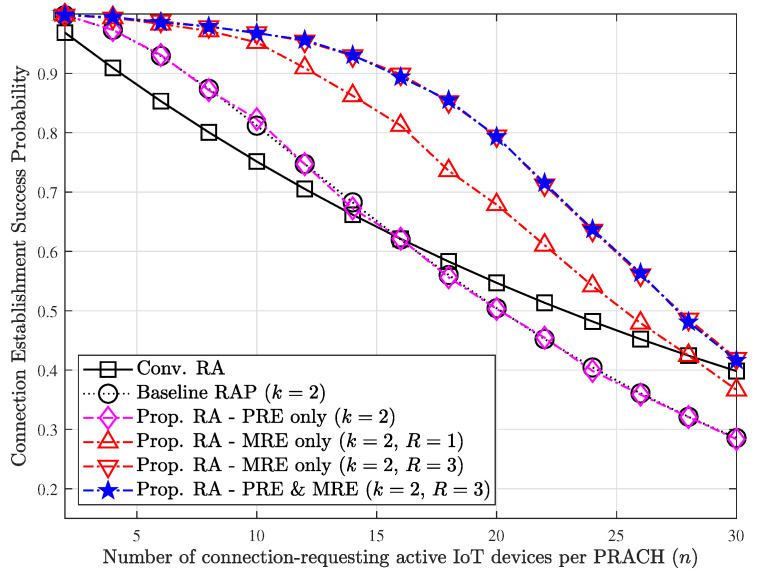
Effect of each of mechanisms on the connection establishment success probability.

Figure 6 shows the connection establishment success probability for varying *n* under the same simulation setup with that of Figure 5. In all cases, the success probability decreases as *n* increases since the occurrence of collisions naturally increases. Note that the RAP scheme presents worse performance compared to the conventional RA scheme when the load is heavy, which is already presented side effect. When we consider our proposed technique with exploiting PRE mechanism only, it is found that the performance gain from the viewpoint of success probability cannot be expected since the PRE mechanism affects the improvement of the resource efficiency only. On the contrary, when we consider our proposed technique exploiting the MRE mechanism regardless of applying PRE or not, the success probability is found to be improved. Considering the benefit from the success probability perspective, slight loss in the resource efficiency may be acceptable according to the requirements.

**Figure 7:**
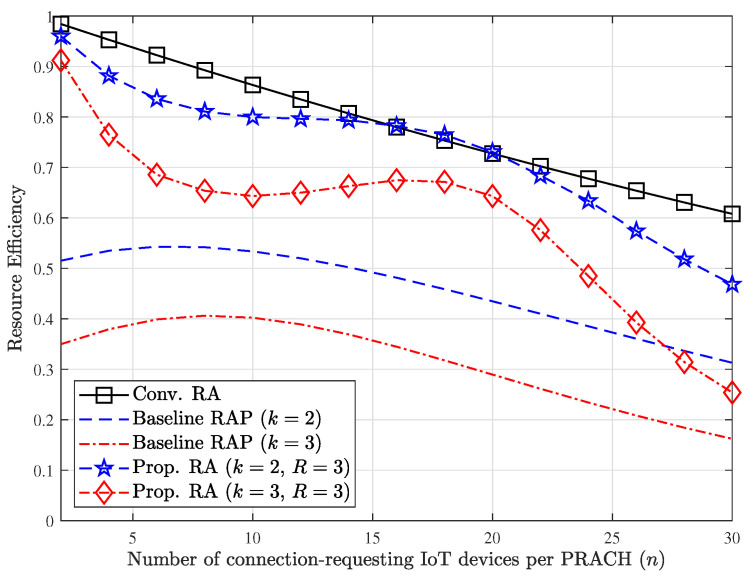
Comparison of resource efficiency for varying *n* when M=32.

Figure 7 shows a comparison between the baseline schemes and our proposed technique from the viewpoint of the resource efficiency for varying *n* when M=32. In case of the proposed RA technique, both redundancy elimination mechanisms are fully utilized, and R=3. It is obvious that two redundancy elimination mechanisms applied to our proposed technique are definitely effective to boost up the resource efficiency compared to the RAP scheme. However, slight degradation in the resource efficiency is mostly inevitable, compared to the conventional RA scheme.

**Figure 8:**
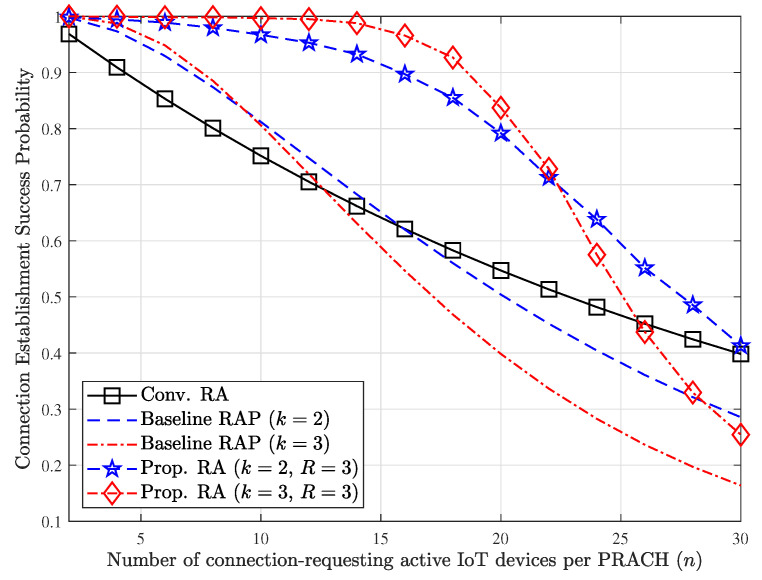
Comparison of connection establishment success probability for varying *n* when M=32.

Figure 8 shows a comparison between the baseline schemes and the proposed RA technique in terms of the connection establishment success probability for varying *n* under the same simulation setup with that of Figure 7. With the baseline RAP scheme, the success probability can be slightly improved compared to the conventional RA scheme within a specific operating region. But, the amount of performance gain steeply decreases as *n* increases since it was reported that increase in the value of *k* causes additional congestion in PRACH [26]. Thanks to our proposed RA technique, however, the success probability can be dramatically improved compared to all baseline schemes. Even though our proposed technique can provide high success probability, the performance degradation beyond a certain point cannot be avoidable. Thus, careful setting in the value of *k* is required jointly considering another aspect such as resource efficiency presented in Figure 7.

## 6. Conclusions

This article newly proposed a resource-efficient parallelized random access (RePRA) technique, which aims to provide resource-efficiently reliable connection establishment performance in cellular-based IoT networks. In our proposed technique, each IoT device simultaneously attempts multiple RAs in parallel to exploit diversity effect during the RA procedure, and the BS handles the inefficient resource utilization with two newly proposed redundancy elimination mechanisms: (1) preamble redundancy elimination (PRE) and (2) message redundancy elimination (MRE). Through extensive simulations, we verified the effectiveness of each mechanism for improving the performances from the viewpoint of radio resource efficiency and the connection establishment success probability. With our proposed technique, the improved connection establishment performance can be exploited with reasonable resource efficiency under a dual-preamble setting, i.e., k=2. If the system requires an extremely high success probability, slight degradation in resource efficiency may be required as a cost. Consequently, we successfully verified the feasibility of our proposed technique for practical use from this research.

## Figures and Tables

**Table 1:** Simulation parameters and values.

Parameters	Values
Number of connection-requesting IoT devices per PRACH (*n*)	2∼30
Number of available preambles (*M*)	32
Number of preambles simultaneously transmitted at Step 1 (*k*)	1∼3
Maximum iteration rounds of the MRE process (*R*)	1∼3

## Data Availability

Not applicable.

## Abbreviations

The following abbreviations are used in this manuscript:

5G	Fifth Generation
6G	Sixth Generation
XR	Extended Reality
AI	Artificial Intelligence
IoT	Internet of Things
AMQP	Advanced Message Queuing Protocol
MQTT	Message Queuing Telemetry Transport
NB-IoT	Narrow Band Internet-of-Things
LTE	Long Term Evolution
NR	New Radio
BS	Base Station
RA	Random Access
RePRA	Resource-efficient Parallelized Random Access
PRACH	Physical Random Access Channel
RAP	Random Access Parallelization
PRE	Preamble Redundancy Elimination
MRE	Message Redundancy Elimination
RAR	Random Access Response
RAPID	Random Access Preamble Index
UG	Uplink Grant
PDP	Power Delay Profile
PDZ	Preamble Detection Zone
